# Association between low eosinophil count and acute bacterial infection, a prospective study in hospitalized older adults

**DOI:** 10.1186/s12877-023-04581-y

**Published:** 2023-12-13

**Authors:** Léa Mésinèle, Tom Pujol, Nicoletta Brunetti, Marie Neiss, Christophe Trivalle, Cecile Souques, Nadège Houenou-Quenum, Sébastien Verdier, Pauline Simon, Anne-Laure Vetillard, Julie Houdre, Rocco Collarino, Morgane Mary, Jean-Sébastien Vidal, Jean-Emmanuel Kahn, Magali Guichardon, Emmanuelle Duron, Edouard Baudouin

**Affiliations:** 1 Service Hospitalo-Universitaire de gériatrie. Assistance Publique-Hôpitaux de Paris, Hôpitaux Universitaires Paris- Saclay, Hôpital Paul-Brousse- Villejuif FR, 12 Avenue Paul Vaillant Couturier, Villejuif, 94800 France; 2 Département de Gériatrie, Assistance Publique-Hôpitaux de Paris, Hôpital Broca, Paris, France; 3 Université Paris Descartes, INSERM, Paris, France; 4 Department of Internal Medicine, APHP, Ambroise Paré Hospital, Université de Versailles-Saint- Quentin en Yvelines, Yvelines, France; 5 CESP, Team MOODS, Université Paris-Saclay, UVSQ, Le Kremlin-Bicêtre, France

**Keywords:** Acute bacterial Infection, Eosinopenia, Diagnosis

## Abstract

**Background:**

The incidence of sepsis increases significantly with age, including a high incidence of bacterial infection in the old adults. Eosinopenia and the CIBLE score have been proposed in critically ill adults and in internal medicine wards. This study aimed to assess whether a low eosinophil count was associated with acute bacterial infection among hospitalized older adults, and to find the most efficient eosinophil count cut-off to differentiate acute bacterial infection from other inflammatory states.

**Methods:**

This was a prospective study from July 2020 to July 2022 in geriatric wards of the University Paul Brousse Hospital (Villejuif, France) including patients aged of 75 y/o or over suffering from fever or biological inflammation. Acute bacterial infection was assessed using biological identification and/or clinical and radiological data.

**Results:**

A total of 156 patients were included. Eighty-two (53%) patients suffered from acute bacterial infection (mean age (SD) 88.7 (5.9)). Low eosinophil count was independently associated with acute bacterial infection: OR [CI95%] 3.03 [1.04–9.37] and 6.08 [2.42–16.5] for eosinophil count 0–0.07 G/L and 0.07–0.172 G/L respectively (vs. eosinophil count > 0.172 G/L). Specificity and sensitivity for eosinophil count < 0.01 G/L and CIBLE score were 84%-49% and 72%-62%, respectively with equivalent AUCs (0.66 and 0.67).

**Conclusion:**

Eosinophil count < 0.01 G/L is a simple, routinely used and inexpensive tool which can easily participate in antibiotic decisions for older adults. Further studies are needed to assess clinical benefits.

**Trial registration:**

The study was registered at Clinical trial.gov (NCT04363138–23/04/2020).

**Supplementary Information:**

The online version contains supplementary material available at 10.1186/s12877-023-04581-y.

## Introduction

The incidence of sepsis increases significantly with age: less than 5/1000 when aged from 50 to 54 years old (y/o) vs. more than 25/1000 when aged 85 y/o and older [1]. Moreover, incidence of bacterial infection also increases with age: in the old adults: 1425.51/100 000 person-year, on 550 432 subjects over 4 years [2].

Diagnosis of acute bacterial infectious diseases can be challenging in hospitalized older adults. Indeed, atypical clinical presentations, such as the absence of fever, may occur in well documented infections in 20–30% of patients [3]. Furthermore, pathogens are distinct from those in younger patients with a high prevalence of hospital acquired infections [4–8]. Finally, commonly accepted biological markers (procalcitonin (PCT) or C reactive protein (CRP)) do not perform as accurately in older adults for bacterial infection diagnosis [9–12]. Thus, without bacterial documentation, no unique biological test can conclude to a bacterial infection [13, 14]. Other biological markers would be useful for diagnosing bacterial infection as antibiotic adverse events in older adults are more frequent and severe (such as delirium, falls and Clostridium difficile infection) [15]. It has been suggested that eosinopenia may occur during acute infection as a result of specific chemotactic agents being released [16].

In fact, a cohort study of 138 patients (mean age (standard deviation): 71.8 (20.8) y/o), eosinophil count < 0.04 G/L when the white blood cell count (WBC) was > 10 G/L showed a specificity of 100% and a sensitivity of 64% [17] for bacterial infections. Furthermore, a composite score (CIBLE score) that includes age, CRP, temperature, chronic obstructive pulmonary disease (COPD) and eosinophils/granulocytes count ratio, was found to predict bacterial infection when > 87, with a 72% sensitivity and a 77% specificity (190 patients, 73.5 (18.2) y/o) [18]. Thus eosinophil count drop, described in other inflammatory states because of eosinophil migration to tissue [19], may also be a marker of acute bacterial infection in older adults.

The goal of this study was to assess whether a low eosinophil count was associated with acute bacterial infection among old adults suffering from fever or inflammation. The secondary objective was to find the eosinophil count cutoff that most effectively differentiate acute bacterial infection from other inflammatory states.

## Methods

### Study design

This is a prospective study from July 2020 to July 2022 in the geriatric acute and rehabilitation wards of Paul Brousse University. The study was approved by an independent ethics committee (Comité de protection des personnes Sud Est V, Grenoble, France: 0-GERO-01, 05/14/2020) and was supported by Gérond’if (Gérontopôle d’Ile de France) (N°IDRCB 2020-A00301-38). All included participants were informed and did not oppose to the study as recommended by French ethics authorities. The study was registered at Clinical trial.gov (NCT04363138) and follows STROBE recommendations (additional file 1).

Inclusion criteria were age ≥ 75 years and fever defined by temperature ≥ 38 °C or biological inflammation: WBC > 10 G/L and CRP > 20 mg/L [18]. Non-inclusion criteria were patients under legal protection or already included in another protocol, patients suffering from any pathologies and treatments known to modify eosinophil cell count (asthma, human immunodeficiency virus infection, malignant hematologic diseases, parasite infection, stroke occurring less than one month before inclusion [20], eosinophilic granulomatous vasculitis, corticosteroid treatment, chemotherapy/immunosuppressive treatments, antibiotics less than one week before inclusion, SARS-CoV-2 infection [21]). Patients suffering from asymptomatic bacteriuria were also excluded. Patients were included only once if they had several fever or biological inflammation episodes during their hospitalization.

### Patients

The following clinical characteristics were collected : age, sex, Charlson comorbidity index (0–29; a score > 4 predicts a one-year mortality risk of 85%) [22], chronic kidney failure defined by a Glomerular Filtration Rate (GFR) < 50 mL/min estimated by CKD-EPI equation [23], severe malnutrition (defined as a BMI < 20 kg/m^2^, ≥ 10% weight loss in a month or ≥ 15% weight loss in 6 months or ≥ 15% of the usual weight before the onset of the disease or serum albumin < 30 g/L) [24], polypharmacy (defined by ≥ 5 treatments), disability assessed by the Activity of Daily Life (ADL) scale (score 0–6, the higher the better) [25], and cognitive function assessed by the Mini Mental State Examination (MMSE) (score 0–30, the higher the better) [26]. Other frequent comorbidities were recorded: major depressive disorder according to DSM-5, hip fracture, atrial fibrillation and treated hypothyroidism.

The following biological data were collected WBC count (laboratory standards: 4–10 G/L), hemoglobin level (13–17 g/dL) and platelet count (150–450 G/L), CRP (< 4 mg/L), serum albumin level (32–46 g/L). Procalcitonin was not available on routine care. Blood count was analyzed by robot Sysmex-XN 1500 and data were collected from medical records.

### Primary outcome

The primary outcome was to compare eosinophil count between two groups: patients with acute bacterial infection and patients without bacterial infection.

Diagnoses of acute bacterial infection were made according to the French Society of Infectious Diseases based on clinical symptoms, imaging data, and microbiological identification such as blood culture, urinalysis and urine culture, polymerase chain reaction (PCR), or stool culture.

Pneumonia diagnosis was based on a physical examination (dyspnea, peripheral oxygen saturation < 95%, or productive cough, with abnormal breath sounds at examination) and radiological confirmation (infiltrate at chest X-ray) if clinical examination was doubtful [27]. Urinary tract infection diagnosis was based on urinary symptoms (frequent urination, dysuria, lower back pain, abdominal pain) and bacteriological confirmation: pyuria > 10^4^/mL with urinary bacterial count > 10^3^ UFC/mL); cholangitis diagnosis was based on physical examination, abnormal liver function test and imaging (abdominal ultrasound or CT scan); diverticulitis diagnosis was based on physical examination and abdominal CT scan; osteitis diagnosis was based on physical examination and CT scan; bacteremia was defined by a positive blood culture.

Inflammation not related to bacterial infections included viral, neoplastic, and other inflammation. Viral infections were diagnosed by PCR (upper respiratory tract sample, cerebrospinal fluid sample). Neoplastic diagnosis was based on physical, radiological and cytopathology confirmation. All other pathologies with biological inflammation diagnosis were based on examination and imaging data.

### Secondary outcome

Sensitivity, specificity and AUC (area under the curve) on bacterial diagnosis of several eosinophil counts cutoff were compared: < 0.04 G/L [28], < 0.01 G/L [17, 29] and eosinophil*1000/neutrophil count < 4 compared to CIBLE score > 87.

### Statistical analysis

A total of 156 subjects needed to be included as calculated with the following parameters: sensitivity 72%, specificity 77%, delta of 0.12, and prevalence of 65%, based on the Bouldoires’ study [18].

Participants’ data are presented as mean and standard deviation (SD) for continuous variables and count (percentage) for categorical variables. T-tests were used for continuous variables and chi-squared tests or Fisher’s exact tests were used for categorical variables. The *p*-values were included for information purposes, only to assess the importance of any difference. Missing values and their distribution in the 2 groups were assessed. Because missing values represented < 2% of the data and were balanced between the 2 cohorts, no specific strategy was necessary. A stepwise multivariate analysis was performed to assess independent variables associated with bacterial infection diagnosis. To avoid overestimation, a conservative approach was used: all variables with p < 0.10 on univariate analysis and all clinically relevant variables from the literature were included. Eosinophil count was transformed to an ordered qualitative variable according to quartiles. Finally, Delong’s tests were used out to compare the areas under the curve (AUCs) of the different values studied.

## Results

### Population description

One hundred fifty-six patients were included between July 2020 and July 2022 (additional file 2). Eighty-two (53%) patients suffered from acute bacterial infection (mean age (SD) 88.7 (5.9) years old). The mean ADL and mean Charlson comorbidity score were respectively 3.8 (2.3) in the bacterial infection group vs. 3.8 (2.2) in the other group (p = 0.96) and 4.5 (3.1) vs. 4 [3] (p = 0.27) respectively. Results are summarized in Table 1 and detailed comorbidities and treatments are summarized in additional files 3 and 4.

**Table 1:** Patients characteristics according to bacterial infection diagnosis

Characteristics	Total n = 156	Acute bacterial infection n = 82 (52.6%)	Non-bacterial inflammation n = 74 (47.4%)	*p*-value
Female (%)	103 (66)	55 (67.1)	48 (64.9)	0.77
Age (SD)	88.8 (5.6)	88.7 (5.9)	89 (5.3)	0.74
ADL (SD)	3.8 (2.3)	3.8 (2.3)	3.8 (2.2)	0.96
BMI (SD)	23.3 (5.8)	23 (6)	23.7 (5.6)	0.53
Missing values (%)	27 (17.3)	16 (19.5)	11 (14.9)	
MMSE (SD)	16.5 (6.8)	17 (6.6)	16 (6.9)	0.47
Missing values (%)	54 (34.6)	31 (37.8)	23 (31.1)	
Charlson comorbidity index (SD)	4.3 (3.1)	4.5 (3.1)	4 (3)	0.27
Severe malnutrition (%)	84 (53.8)	46 (56.1)	38 (51.4)	0.55
Atrial fibrillation, (%)	54 (34.6)	31 (37.8)	23 (31.1)	0.38
Treated hypothyroidism (%)	10 (13.5)	4 (4.9)	14 (9)	0.06
Hip fracture, (%)	25 (16)	10 (12.2)	15 (20.3)	0.17
Number of treatments, (SD)	7 (3.1)	7.3 (3.4)	6.7 (2.8)	0.23
Deceased within 30 days, (%)	17 (10.9)	10 (12.2)	7 (9.5)	0.584

Note: Data presented as mean (SD) or count (%)

Abbreviations: ADL, activity daily living; BMI, body mass index; MMSE, mini mental state examination; SD, Standard Deviation

### Main diagnosis

**Fig. 1:**
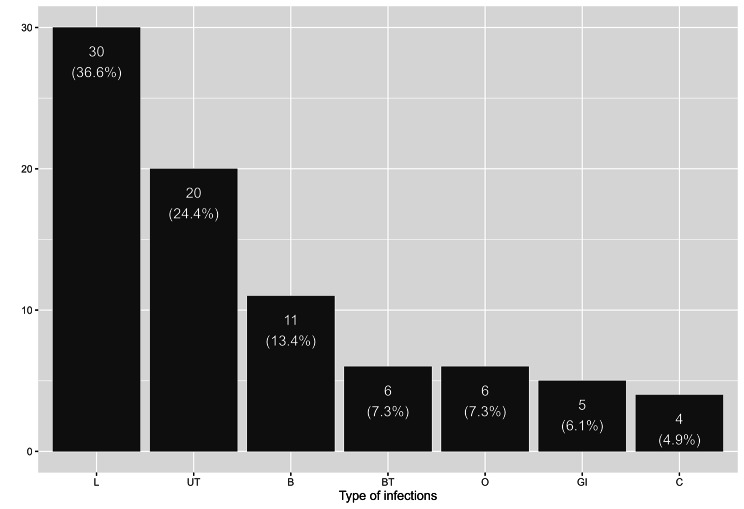
Histogram of infection sites in the acute bacterial infection group. Legends: L, Lungs; UT, Urinary Tract; B, Bacteriemia; BT, Biliary Tract; O, Osteitis/Spondylodiscitis; GI, Gastrointestinal; C, Cutaneous

Overall, 112 (72%) patients were included because of biological inflammation and 88 (56%) because of fever, 45 (29%) patients because of both. In the acute bacterial infection group, 30 (37%) patients suffered from pulmonary infection including COPD exacerbation and 20 (24%) from urinary tract infection (Fig. 1). Of the 82 acute bacterial infections, 40 had pathogen identification, 24 diagnoses were made using clinico-radiological data and 18 on clinical argument alone. Details of pathogens and sites are provided in additional file 3. In the non-bacterial inflammation group, 34 (44%) patients suffered from inflammatory conditions (thrombosis, chondrocalcinosis, myocardial infarction, vascularitis), 11 (14%) from viral infections and 11 (14%) from neoplasm. For 6 (8%) patients, no diagnosis was found: elevated CRP or fever of unknown origin.

### Eosinophil count association with acute bacterial Infection

The mean eosinophil count was 0.1 (0.2) G/L, 0.1 (0.1) G/L and 0.2 (0.2) G/L in the acute bacterial group and non-bacterial diagnosis group respectively (p < 0.001). Eosinophil count < 0.04 G/L, eosinophil count < 0.01 G/L and eosinophil/neutrophil count < 4 were more frequent in the acute bacterial group than in the non-bacterial diagnosis group (56.1% vs. 25.7%, p < 0.001; 48.8% vs. 16.2%, p < 0.001; and 52.4% vs. 21.6%, p < 0.001 respectively). Biological data are presented in Table 2.

A CIBLE score > 87 was more frequent in group 1 vs. group 2 (62.2% vs. 28.4%, p < 0.001).

**Table 2:** Patients’ biological characteristics and CIBLE score > 87 according to acute bacterial infection diagnosis

Biological characteristics	Total n = 156	Acute bacterial infection n = 82 (52.6%)	Non-bacterial inflammation n = 74 (47.4%)	*p*-value
Hemoglobin, g/dL (SD)	11.6 (1.9)	11.7 (1.9)	11.6 (1.9)	0.63
Platelet count, G/L (SD)	289.1 (114.3)	272.7 (118.1)	307.1 (107.8)	0.06
Serum albumin, g/L (SD)	29.4 (5.3)	29.2 (5.5)	29.7 (5.1)	0.56
CRP, mg/L (SD)	103.9 (86.6)	114.1 (87.3)	92.5 (84.9)	0.12
Missing values (%)	1 (0.6)	0	1 (1.4)	
Eosinophil count, G/L (SD)	0.1 (0.2)	0.1 (0.1)	0.2 (0.2)	< 0.001
Neutrophil count, G/L (SD)	9.6 (4.7)	10.7 (5.8)	8.4 (2.5)	0.001
Lymphocyte count, G/L (SD)	1.3 (0.7)	1.1 (0.6)	1.4 (0.7)	0.007
Eosinophil < 0.01 G/L (%)	52 (33.3)	40 (48.8)	12 (16.2)	< 0.001
Eosinophil < 0.04 G/L (%)	65 (41.7)	46 (56.1)	19 (25.7)	< 0.001
Eosinophil * 1000/PMN < 4 (%)	59 (37.8)	43 (52.4)	16 (21.6)	< 0.001
CIBLE score > 87 (%)	72 (46.2)	51 (62.2)	21 (28.4)	< 0.001

Note: Data presented as mean (SD) or numbers (%)

Abbreviations: CRP, C-reactive protein; SD, Standard Deviation

In multivariate analysis, eosinophil count (ref: > 0.172 G/L) was independently associated with bacterial diagnosis: eosinophil count (0.07–0.172) OR 6.08 [2.42–16.5], (0–0.07) OR 3.03 [1.04–9.37] (Table 3).

**Table 3:** Logistic regression of bacterial diagnosis as dependent variable

Characteristics	OR [95% CI]	*p*-value
Age	1 [0.94–1.08]	> 0.9
Sex (ref = Male)	1.08 [0.49–2.42]	0.8
Treated hypothyroidism	0.21 [0.03–0.97]	0.07
Fever at inclusion	4.43 [1.97–10.5]	< 0.001
Neutrophil count (G/L)	1.25 [1.11–1.43]	< 0.001
Eosinophil count (G/L) (ref: > 0.172)		
]0.07–0.172]	6.08 [2.42–16.5]	< 0.001
[0–0.07]	3.03 [1.04–9.37]	0.047

Abbreviations: CI, Confidence Interval; OR, Odds Ratio

Eosinophil count < 0.01 G/L had the best specificity compared to the CIBLE score > 87 (84% vs. 72%) but a lower sensitivity (49% vs. 62%) with a comparable AUC (0.67 vs. 0.66; p = 0.87). All areas under the curve (AUC), sensitivities and specificities are presented in Table 4.

**Table 4:** Area under the curve (AUC), sensitivity and specificity of the biological variables associated with acute bacterial infections

	AUC [IC95%]	Sensitivity	Specificity	*p*-value
**CIBLE score > 87**	0.67 [0.60–0.74]	62%	72%	
**Eosinophil count < 0.01 G/L**	0.66 [0.59–0.73]	49%	84%	0.87
**Eosinophil count < 0.04 G/L**	0.65 [0.58–0.73]	56%	74%	0.64
**Eosinophil count < 0.01 G/L and CIBLE score > 87**	0.66 [0.59–0.72]	43%	89%	0.76
**Eosinophil count < 0.01 G/L or CIBLE score > 87**	0.67 [0.59–0.74]	67%	66%	0.90
**Eosinophil/neutrophil count < 4**	0.65 [0.58–0.72]	52%	78%	0.66
**Lymphocyte count (G/L)**	0.62 [0.53–0.71]	74%	38%	0.37
**Neutrophil count (G/L)**	0.61 [0.52–0.70]	59%	57%	0.30
**CRP (mg/L)**	0.42 [0.33–0.51]	65%	49%	0.03

Abbreviations: AUC, Area Under the Curve

## Discussion

This study shows that a low eosinophil count is independently associated with acute bacterial infection among hospitalized older adults: OR 3.03 [1.04–9.37] for eosinophil count 0–0.07 G/L compared to eosinophil count > 0.172 G/L. Eosinophil count < 0.01 G/L had better specificity (84%) than CIBLE score > 87, eosinophil count < 0.04 G/L, eosinophil/neutrophil ratio and CRP 72%, 74%78% and 49% respectively. While using both CIBLE score > 87 and eosinophil together improved specificity (89%), it did not improve AUC and decreased sensitivity (43%), at the coast of complicating the use in routine practice. Although the sensitivity of eosinophil count < 0.01 G/L was the lowest (49%), comparable AUCs were observed except for CRP, for which it was the lowest.

This result correlates with a prospective study (96 patients, mean age (SD): 64 [21] years old)), in which patients suffering from bacterial infection with eosinophil count < 0.01 G/L had a faster eosinophil normalization than CRP [19]. More recently, a retrospective study including 197 patients of an age closer to that ouf our study (mean age (SD): 89.6 (5.7)), found that persistent eosinophil count < 0.1 G/L between day 2 and day 4, was associated with in-hospital mortality (HR: 8.9 [3.46–22.9]) [30]. However, eosinophil count < 0.04 G/L combined with WBC > 10 G/L has been studied in an internal medicine ward on 138 patients aged of 71.8 (29.9) y/o, and had high specificity (100%) with a sensitivity of 64% [17].This result was not confirmed in our study, probably because WBC > 10 G/L and a CRP > 20 mg were inclusion criteria and included patients were older (88.8 (5.6) y/o compared to 71.8 y/o). This may be significant as inflammatory response changes with the ageing process [31]. Moreover, a meta-analysis [28] showed that a threshold of 0.02 G/L had better specificity for sepsis diagnosis (0.83 (0.80–0.85)) than a threshold of 0.04 G/L (0.75 (0.69–0.80)). However, like our study, this meta-analysis found low sensitivities. This is relevant because a low eosinophil count (e.g. < 0.01 G/L) may not be useful as a positive diagnosis marker but rather as a tool to exclude bacterial infection diagnosis.

To our knowledge, no study provided biological hypothesis to explain low eosinophil count specificity to acute bacterial infections. However, it may be partly explained by high recruitment in the tissues, secondary to chemoattractant released. Indeed, after being recruited, they improve membrane permeability allowing passage for other immune cells [32].

We acknowledge that this study has some limitations. First, it takes into account only one eosinophil dosage which has been shown to vary rapidly during the day [33]. However, as this is a prospective study, the biological samples were taken during standard care. Thus, they were routinely done at the same time and/or under the same circumstances. Second, some data are missing, mainly MMSE score, but to our knowledge, no relationship between cognitive disorders and low eosinophil count has been reported. Third, while COPD is a frequent pathology among the old adults (47.7% in the 75–80 y/o) [34], only 17 (10.9%) subjects with COPD were included. This may explain the lower specificity of the CIBLE score > 87. However, as corticosteroid therapy is a frequent COPD treatment and is known to alter eosinophil count [35, 36], this would have biased the results.

Nonetheless, to the best of our knowledge, this is first prospective study to evaluate eosinophil count as a marker for acute bacterial infection in hospitalized old adults.

Overall, we found that the use of eosinophil count < 0.01 G/L, along with other clinical and biological parameters, can be of interest to postpone antibiotic treatments and further investigations. This result is not intended to be used as diagnosis tool nor to replace gold standard. However, compared to the CIBLE score or other scores that need specific calculation tools, this is a daily routine exam and may be used to avoid over prescription and iatrogenic effects in this population [37, 38].

## Conclusion

In this prospective monocentric study, conducted on 156 older inpatients in geriatric departments, a low eosinophil count was independently associated with the diagnosis of acute bacterial infection. Eosinophil < 0.01 G/L is a simple, routinely used and inexpensive tool that can easily participate in the medical decision to postpone antibiotic treatment. Further studies are needed to assess the clinical benefits in a larger population.

### Electronic supplementary material

Below is the link to the electronic supplementary material.

Supplementary Material 1

Supplementary Material 2

Supplementary Material 3

Supplementary Material 4

## Data Availability

The datasets used and analyzed during the current study are available from the corresponding author on reasonable request.

## Abbreviations

ADL: Activity of Daily Life
AUC: Area Under the Curve
BMI: Body Mass Index
CRP: C-reactive protein
GFR: Glomerular Filtration Rate
MMSE: Mini Mental Status Examination
PCR: Polymerase Chain Reaction
PCT: Procalcitonin
WBC: White blood cells
