# Supplementary figures and images for: Association between low eosinophil count and acute bacterial infection, a prospective study in hospitalized older adults
Source: BMC Geriatr. 2023 Dec 13;23:852. doi: 10.1186/s12877-023-04581-y (PMC10720062; doi:10.1186/s12877-023-04581-y)

**Additional file 2** : Flow chart


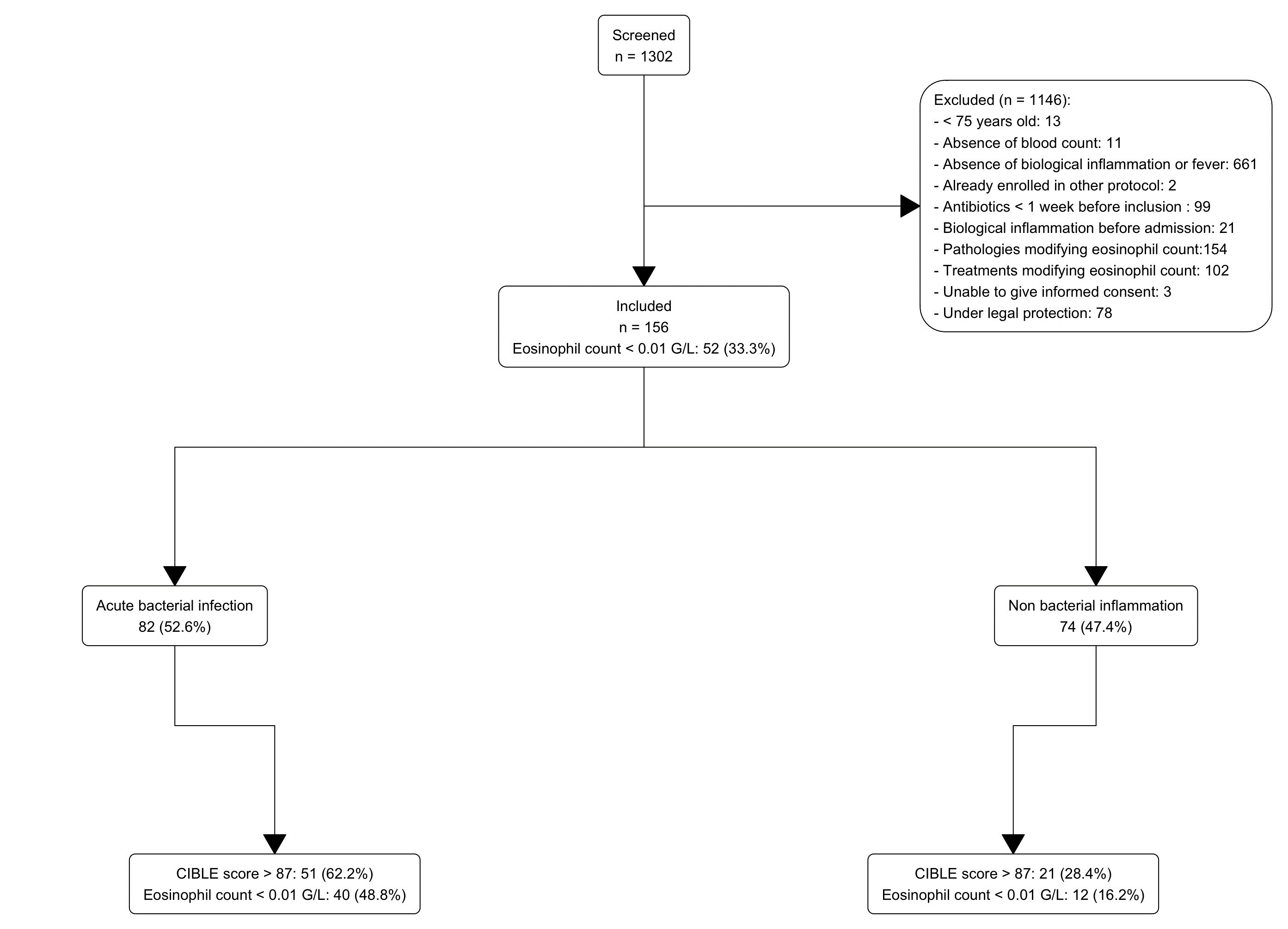

Supplement: Supplementary file 2 — Supplementary Material 2 [file 12877_2023_4581_MOESM2_ESM.docx]
