# Supplementary material for: Association between low eosinophil count and acute bacterial infection, a prospective study in hospitalized older adults
Source: BMC Geriatr. 2023 Dec 13;23:852. doi: 10.1186/s12877-023-04581-y (PMC10720062; doi:10.1186/s12877-023-04581-y)
Supplement: Supplementary file 3 — Supplementary Material 3 [file 12877_2023_4581_MOESM3_ESM.docx]

| **Additional file 3**: Detailed comorbidities according to group | | | | |
| --- | --- | --- | --- | --- |
| **Characteristics** | **Total**  **n = 156** | **Acute bacterial infection**  **n = 82 (52.6%)** | **Non-bacterial inflammation**  **n = 74 (47.4%)** | **p-value** |

| Chronic heart failure (%) | 68 (43.6) | 36 (43.9) | 32 (43.2) | 0.93 |
| --- | --- | --- | --- | --- |
| Atrial fibrillation (%) | 54 (34.6) | 31 (37.8) | 23 (31.1) | 0.38 |
| Ischemic heart failure (%) | 37 (23.7) | 22 (26.8) | 15 (20.3) | 0.34 |
| Peripheral vascular disease (%) | 22 (14.1) | 8 (9.8) | 14 (18.9) | 0.10 |
| COPD (%) | 17 (10.9) | 11 (13.4) | 6 (8.1) | 0.29 |
| History of stroke (%) | 43 (27.6) | 23 (28) | 20 (27) | 0.89 |
| Hemiplegia (%) | 3 (1.9) | 2 (2.4) | 1 (1.4) | 0.99 |
| History of major depressive disorder | 48 (30.8) | 26 (31.7) | 22 (29.7) | 0.79 |
| Diabetes (%) | 35 (22.4) | 23 (28) | 12 (16.2) | 0.08 |
| Chronic kidney failure (%) | 55 (35.3) | 28 (34.1) | 27 (36.5) | 0.76 |
| Peptic ulcer (%) | 11 (7.1) | 4 (4.9) | 7 (9.5) | 0.26 |
| Liver disease (%) | 6 (3.8) | 1 (1.2) | 5 (6.8) | 0.10 |
| Solid cancer (%) | 41 (26.3) | 25 (30.5) | 16 (21.6) | 0.21 |
| Connective tissue diseases (%) | 6 (3.8) | 3 (3.7) | 3 (4.1) | 0.99 |
| Abbreviations: COPD: Chronic obstructive pulmonary disease. CRP: C-reactive protein | | | | |
