# Supplementary material for: Association between low eosinophil count and acute bacterial infection, a prospective study in hospitalized older adults
Source: BMC Geriatr. 2023 Dec 13;23:852. doi: 10.1186/s12877-023-04581-y (PMC10720062; doi:10.1186/s12877-023-04581-y)
Supplement: Supplementary file 4 — Supplementary Material 4 [file 12877_2023_4581_MOESM4_ESM.doc]

| **Additional file 4:** Detailed treatments according to group | | | | |
| --- | --- | --- | --- | --- |
| Characteristics | **Total**  **n = 156** | **Acute bacterial infection**  **n = 82 (52.6%)** | **Non-bacterial inflammation**  **n = 74 (47.4%)** | **p-value** |
| Antiplatelet/anticoagulant | **98 (62.8)** | **56 (68.3)** | **42 (56.8)** | **0.14** |
| Benzodiazepine or hypnotic treatment | 62 (39.7) | 32 (39) | 30 (40.5) | 0.85 |
| Pregabalin/ gabapentin | 17 (10.9) | 10 (12.2) | 7 (9.5) | 0.58 |
| ACE inhibitor/ ARBs | 38 (24.4) | 17 (20.7) | 21 (28.4) | 0.27 |
| Proton pump inhibitor | 76 (48.7) | 41 (50) | 35 (47.3) | 0.74 |
| Abbreviations: ACE, angiotensin-converting enzyme; ARBs, Angiotensin II receptor blockers. | | | | |
